# The genome sequence of the Mottled Grey, *Colostygia multistrigaria* (Haworth, 1809) (Lepidoptera: Geometridae)

**DOI:** 10.12688/wellcomeopenres.25410.1

**Published:** 2025-12-23

**Authors:** Liam M. Crowley, Cian D Williams, University of Oxford and Wytham Woods Genome Acquisition Lab, Darwin Tree of Life Barcoding Collective, Wellcome Sanger Institute Tree of Life Management, Samples and Laboratory team, Wellcome Sanger Institute Scientific Operations: Sequencing Operations, Wellcome Sanger Institute Tree of Life Core Informatics team, Tree of Life Core Informatics collective, Darwin Tree of Life Consortium

**Affiliations:** 1 Department of Biology, University of Oxford, Oxford, England, UK; 2 Department of Zoology, University of Cambridge, Cambridge, England, UK

**Keywords:** Colostygia multistrigaria; Mottled Grey; genome sequence; chromosomal; Lepidoptera

## Abstract

We present a genome assembly from an individual male *Colostygia multistrigaria* (Mottled Grey; Arthropoda; Insecta; Lepidoptera; Geometridae). The assembly contains two haplotypes with total lengths of 482.72 megabases and 504.67 megabases. Most of haplotype 1 (99.19%) is scaffolded into 31 chromosomal pseudomolecules, including the Z sex chromosome. Haplotype 2 was assembled to scaffold level. The mitochondrial genome has also been assembled, with a length of 17.63 kilobases. This assembly was generated as part of the Darwin Tree of Life project, which produces reference genomes for eukaryotic species found in Britain and Ireland.

## Species taxonomy

Eukaryota; Opisthokonta; Metazoa; Eumetazoa; Bilateria; Protostomia; Ecdysozoa; Panarthropoda; Arthropoda; Mandibulata; Pancrustacea; Hexapoda; Insecta; Dicondylia; Pterygota; Neoptera; Endopterygota; Amphiesmenoptera; Lepidoptera; Glossata; Neolepidoptera; Heteroneura; Ditrysia; Obtectomera; Geometroidea; Geometridae; Larentiinae; *Colostygia*; *Colostygia multistrigaria* (Haworth, 1809) (NCBI:txid1869728)

## Background

The Mottled Grey, *Colostygia multistrigaria*, is a medium-sized (26–31 mm wingspan) moth in the family Geometridae. Its wings are grey mottled with black or brown, with a weak dark central band and chequered markings along the veins. There is variation in pigmentation throughout its range, leading some taxonomists to recognise multiple subspecies, for example subsp. *nebulata* (Duponchel, 1843) and subsp. *olbiaria* (Miller, 1865), which is paler and is restricted to the Iberian Peninsula ( GBIF Secretariat, 2025). Caterpillars are a cryptic brown-grey, with dark longitudinal bands ( Hausmann & Viidalepp, 2012).

It is common and widespread throughout the UK and western Europe ( GBIF Secretariat, 2025). It can be found across woodlands, moorland and heathland where the larvae feed on bedstraw ( *Galium* spp.). Adults are nocturnal, but are attracted to light, and can often be seen flying at dusk. They can be seen on the wing in early spring, most commonly from February to May ( GBIF Secretariat, 2025), although there is some evidence that their phenology may be changing, possibly driven by climate change ( Riley, 1990). However, responses of this species’ life-history and population dynamics to climatic events are complex ( Palmer *et al.*, 2017), and further research is needed to fully assess possible impacts.

This genome sequence was determined as part of the Darwin Tree of Life project, and is also included in Project Psyche, an effort to sequence the genomes of all European Lepidoptera. Recent molecular phylogenetic work has resulted in a well-resolved phylogeny for the European Geometridae, which included four members of *Colostygia*, but not *C. multistrigaria* ( Õunap *et al.*, 2025). This genome sequence will be useful for resolving the taxonomy of this genus, as well as determining the genomic distinctiveness of proposed subspecies. It will also facilitate research into phenological responses to climate change, and evolutionary drivers of variation in pigmentation.

## Methods

### Sample acquisition and DNA barcoding

**Figure 1.**
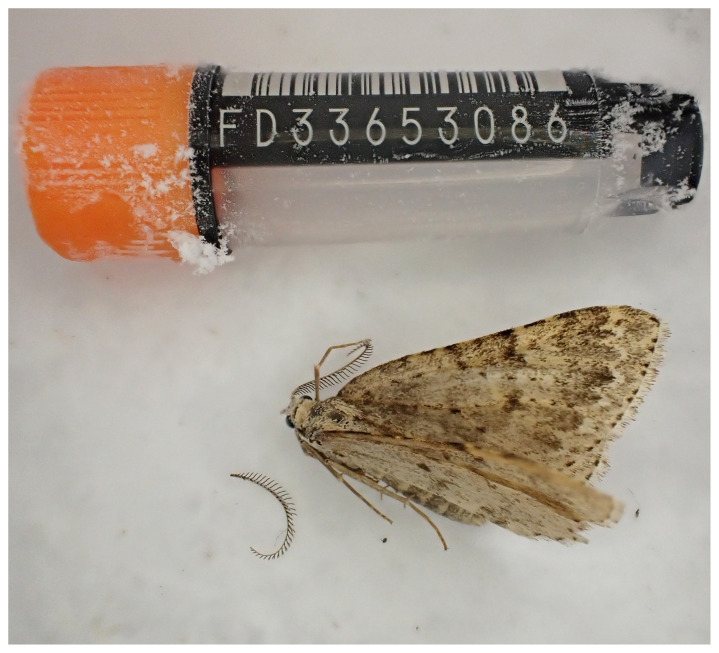
Photograph of the *Colostygia multistrigaria* (ilColMult2) specimen used for genome sequencing.

The specimen used for genome sequencing was an adult male *Colostygia multistrigaria* (specimen ID Ox003351, ToLID ilColMult2; Figure 1), collected from Wytham Woods, Oxfordshire, United Kingdom (latitude 51.772, longitude –1.338) on 2023-03-27. A second specimen was used for Hi-C sequencing (specimen ID Ox003344, ToLID ilColMult1). It was collected from the same location on 2023-03-31. The The specimens were collected and identified by Liam Crowley (University of Oxford). Sample metadata were collected in line with the Darwin Tree of Life project standards described by Lawniczak *et al.* (2022).

The initial identification was verified by an additional DNA barcoding process according to the framework developed by Twyford *et al.* (2024). A small sample was dissected from the specimen and stored in ethanol, while the remaining parts were shipped on dry ice to the Wellcome Sanger Institute (WSI) (see the protocol). The tissue was lysed, the COI marker region was amplified by PCR, and amplicons were sequenced and compared to the BOLD database, confirming the species identification ( Crowley *et al.*, 2023). Following whole genome sequence generation, the relevant DNA barcode region was also used alongside the initial barcoding data for sample tracking at the WSI ( Twyford *et al.*, 2024). The standard operating procedures for Darwin Tree of Life barcoding are available on protocols.io.

### Nucleic acid extraction

Protocols for high molecular weight (HMW) DNA extraction developed at the Wellcome Sanger Institute (WSI) Tree of Life Core Laboratory are available on protocols.io ( Howard *et al.*, 2025). The ilColMult2 sample was weighed and triaged to determine the appropriate extraction protocol. Tissue from the whole organism was homogenised by powermashing using a PowerMasher II tissue disruptor. HMW DNA was extracted using the Automated MagAttract v2 protocol. We used centrifuge-mediated fragmentation to produce DNA fragments in the 8–10 kb range, following the Covaris g-TUBE protocol for ultra-low input (ULI). Sheared DNA was purified by automated SPRI (solid-phase reversible immobilisation). The concentration of the sheared and purified DNA was assessed using a Nanodrop spectrophotometer and Qubit Fluorometer using the Qubit dsDNA High Sensitivity Assay kit. Fragment size distribution was evaluated by running the sample on the FemtoPulse system. For this sample, the final post-shearing DNA had a Qubit concentration of 3.16 ng/μL and a yield of 410.80 ng.

### PacBio HiFi library preparation and sequencing

Library preparation and sequencing were performed at the WSI Scientific Operations core. Prior to library preparation, the DNA was fragmented to ~10 kb. Ultra-low-input (ULI) libraries were prepared using the PacBio SMRTbell® Express Template Prep Kit 2.0 and gDNA Sample Amplification Kit. Samples were normalised to 20 ng DNA. Single-strand overhang removal, DNA damage repair, and end-repair/A-tailing were performed according to the manufacturer’s instructions, followed by adapter ligation. A 0.85× pre-PCR clean-up was carried out with Promega ProNex beads.

The DNA was evenly divided into two aliquots for dual PCR (reactions A and B), both following the manufacturer’s protocol. A 0.85× post-PCR clean-up was performed with ProNex beads. DNA concentration was measured using a Qubit Fluorometer v4.0 (Thermo Fisher Scientific) with the Qubit HS Assay Kit, and fragment size was assessed on an Agilent Femto Pulse Automated Pulsed Field CE Instrument (Agilent Technologies) using the gDNA 55 kb BAC analysis kit. PCR reactions A and B were then pooled, ensuring a total mass of ≥500 ng in 47.4 μl.

The pooled sample underwent another round of DNA damage repair, end-repair/A-tailing, and hairpin adapter ligation. A 1× clean-up was performed with ProNex beads, followed by DNA quantification using the Qubit and fragment size analysis using the Agilent Femto Pulse. Size selection was performed on the Sage Sciences PippinHT system, with target fragment size determined by Femto Pulse analysis (typically 4–9 kb). Size-selected libraries were cleaned with 1.0× ProNex beads and normalised to 2 nM before sequencing.

The sample was sequenced on a Revio instrument (Pacific Biosciences). The prepared library was normalised to 2 nM, and 15 μL was used for making complexes. Primers were annealed and polymerases bound to generate circularised complexes, following the manufacturer’s instructions. Complexes were purified using 1.2X SMRTbell beads, then diluted to the Revio loading concentration (200–300 pM) and spiked with a Revio sequencing internal control. The sample was sequenced on a Revio 25M SMRT cell. The SMRT Link software (Pacific Biosciences), a web-based workflow manager, was used to configure and monitor the run and to carry out primary and secondary data analysis.

### Hi-C

***Sample preparation and crosslinking***

The Hi-C sample was prepared from 20–50 mg of frozen head and thorax tissue of the ilColMult1 sample using the Arima-HiC v2 kit (Arima Genomics). Following the manufacturer’s instructions, tissue was fixed and DNA crosslinked using TC buffer to a final formaldehyde concentration of 2%. The tissue was homogenised using the Diagnocine Power Masher-II. Crosslinked DNA was digested with a restriction enzyme master mix, biotinylated, and ligated. Clean-up was performed with SPRISelect beads before library preparation. DNA concentration was measured with the Qubit Fluorometer (Thermo Fisher Scientific) and Qubit HS Assay Kit. The biotinylation percentage was estimated using the Arima-HiC v2 QC beads.

***Hi-C library preparation and sequencing***

Biotinylated DNA constructs were fragmented using a Covaris E220 sonicator and size selected to 400–600 bp using SPRISelect beads. DNA was enriched with Arima-HiC v2 kit Enrichment beads. End repair, A-tailing, and adapter ligation were carried out with the NEBNext Ultra II DNA Library Prep Kit (New England Biolabs), following a modified protocol where library preparation occurs while DNA remains bound to the Enrichment beads. Library amplification was performed using KAPA HiFi HotStart mix and a custom Unique Dual Index (UDI) barcode set (Integrated DNA Technologies). Depending on sample concentration and biotinylation percentage determined at the crosslinking stage, libraries were amplified with 10–16 PCR cycles. Post-PCR clean-up was performed with SPRISelect beads. Libraries were quantified using the AccuClear Ultra High Sensitivity dsDNA Standards Assay Kit (Biotium) and a FLUOstar Omega plate reader (BMG Labtech).

Prior to sequencing, libraries were normalised to 10 ng/μL. Normalised libraries were quantified again to create equimolar and/or weighted 2.8 nM pools. Pool concentrations were checked using the Agilent 4200 TapeStation (Agilent) with High Sensitivity D500 reagents before sequencing. Sequencing was performed using paired-end 150 bp reads on the Illumina NovaSeq 6000.

### Genome assembly

Prior to assembly of the PacBio HiFi reads, a database of *k*-mer counts ( *k* = 31) was generated from the filtered reads using FastK. GenomeScope2 ( Ranallo-Benavidez *et al.*, 2020) was used to analyse the *k*-mer frequency distributions, providing estimates of genome size, heterozygosity, and repeat content.

The HiFi reads were assembled using Hifiasm in Hi-C phasing mode ( Cheng *et al.*, 2021; Cheng *et al.*, 2022), producing two haplotypes. Hi-C reads ( Rao *et al.*, 2014) were mapped to the primary contigs using bwa-mem2 ( Vasimuddin *et al.*, 2019). Contigs were further scaffolded with Hi-C data in YaHS ( Zhou *et al.*, 2023), using the --break option for handling potential misassemblies. The scaffolded assemblies were evaluated using Gfastats ( Formenti *et al.*, 2022), BUSCO ( Manni *et al.*, 2021) and MERQURY.FK ( Rhie *et al.*, 2020).

The mitochondrial genome was assembled using MitoHiFi ( Uliano-Silva *et al.*, 2023).

### Assembly curation

The assembly was decontaminated using the Assembly Screen for Cobionts and Contaminants ( ASCC) pipeline. TreeVal was used to generate the flat files and maps for use in curation. Manual curation was conducted primarily in PretextView and HiGlass ( Kerpedjiev *et al.*, 2018). Scaffolds were visually inspected and corrected as described by Howe *et al.* (2021). Manual corrections included 66 breaks and 135 joins. This reduced the scaffold count by 5.3% and increased the scaffold N50 by 0.6%. The curation process is described at https://gitlab.com/wtsi-grit/rapid-curation. PretextSnapshot was used to generate a Hi-C contact map of the final assembly.

### Assembly quality assessment

The Merqury.FK tool ( Rhie *et al.*, 2020) was run in a Singularity container ( Kurtzer *et al.*, 2017) to evaluate *k*-mer completeness and assembly quality for both haplotypes using the *k*-mer databases ( *k* = 31) computed prior to genome assembly. The analysis outputs included assembly QV scores and completeness statistics.

The genome was analysed using the BlobToolKit pipeline, a Nextflow implementation of the earlier Snakemake version ( Challis *et al.*, 2020). The pipeline aligns PacBio reads using minimap2 ( Li, 2018) and SAMtools ( Danecek *et al.*, 2021) to generate coverage tracks. It runs BUSCO ( Manni *et al.*, 2021) using lineages identified from the NCBI Taxonomy ( Schoch *et al.*, 2020). For the three domain-level lineages, BUSCO genes are aligned to the UniProt Reference Proteomes database ( Bateman *et al.*, 2023) using DIAMOND blastp ( Buchfink *et al.*, 2021). The genome is divided into chunks based on the density of BUSCO genes from the closest taxonomic lineage, and each chunk is aligned to the UniProt Reference Proteomes database with DIAMOND blastx. Sequences without hits are chunked using seqtk and aligned to the NT database with blastn ( Altschul *et al.*, 1990). The BlobToolKit suite consolidates all outputs into a blobdir for visualisation. The BlobToolKit pipeline was developed using nf-core tooling ( Ewels *et al.*, 2020) and MultiQC ( Ewels *et al.*, 2016), with containerisation through Docker ( Merkel, 2014) and Singularity ( Kurtzer *et al.*, 2017).

## Genome sequence report

### Sequence data

**Figure 2.**
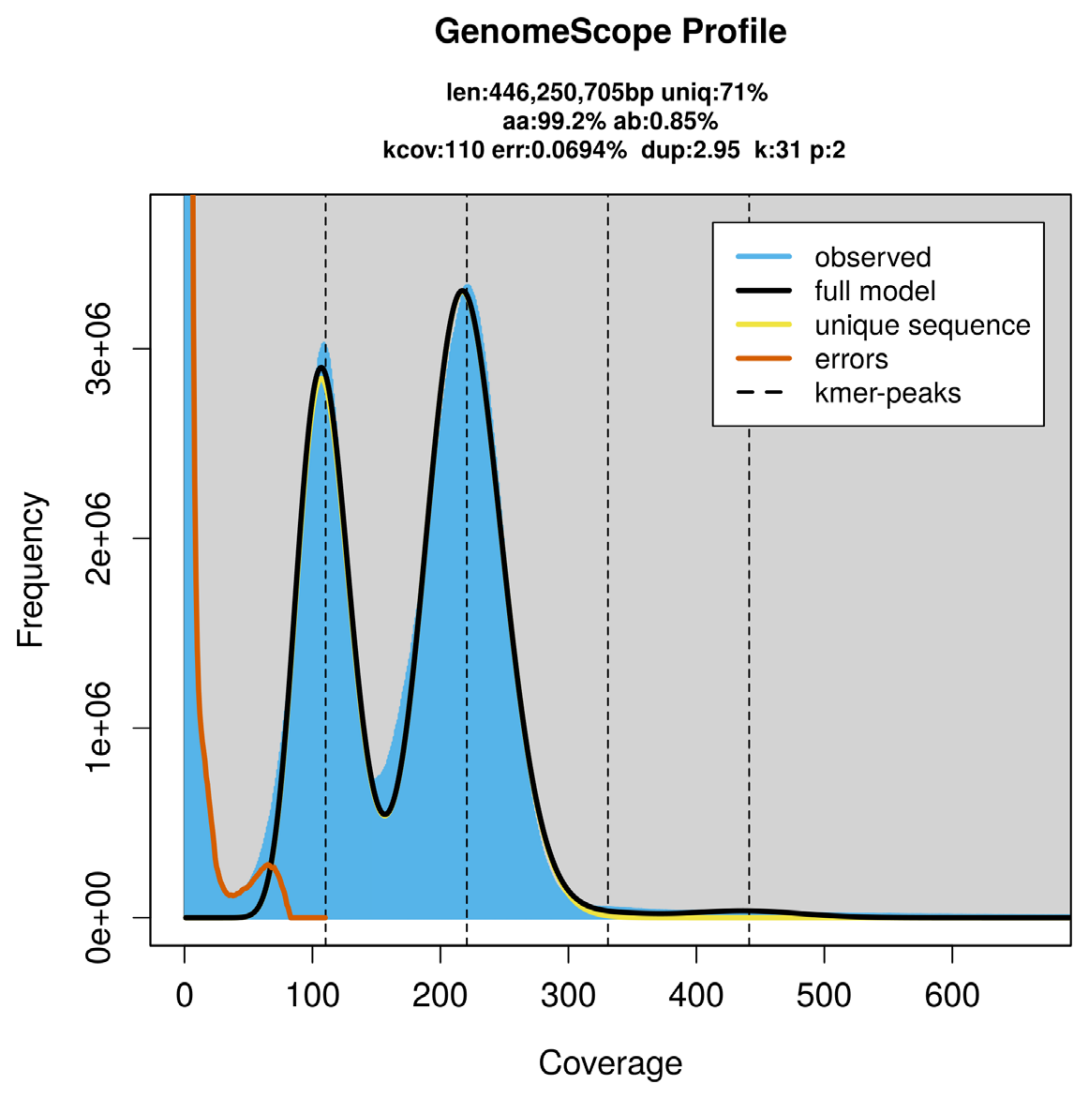
Frequency distribution of *k*-mers generated using GenomeScope2. The plot shows observed and modelled *k*-mer spectra, providing estimates of genome size, heterozygosity, and repeat content based on unassembled sequencing reads.

PacBio sequencing of the *Colostygia multistrigaria* specimen generated 105.06 Gb (gigabases) from 14.79 million reads, which were used to assemble the genome. GenomeScope2.0 analysis estimated the haploid genome size at 446.25 Mb, with a heterozygosity of 0.85% and repeat content of 29.09% ( Figure 2). These estimates guided expectations for the assembly. Based on the estimated genome size, the sequencing data provided approximately 88× coverage. Hi-C sequencing produced 115.64 Gb from 765.83 million reads, which were used to scaffold the assembly. Table 1 summarises the specimen and sequencing details.

**Table 1.** Specimen and sequencing data for BioProject PRJEB84117.

Platform	PacBio HiFi	Hi-C
**ToLID**	ilColMult2	ilColMult1
**Specimen ID**	Ox003351	Ox003344
**BioSample (source individual)**	SAMEA113425853	SAMEA113425846
**BioSample (tissue)**	SAMEA113426053	SAMEA113426038
**Tissue**	whole organism	head and thorax
**Instrument**	Revio	Illumina NovaSeq 6000
**Run accessions**	ERR15170320; ERR14121444	ERR14125370
**Read count total**	14.79 million	765.83 million
**Base count total**	105.06 Gb	115.64 Gb

### Assembly statistics

The genome was assembled into two haplotypes using Hi-C phasing. Haplotype 1 was curated to chromosome level, while haplotype 2 was assembled to scaffold level. The final assembly has a total length of 482.72 Mb in 124 scaffolds, with 240 gaps, and a scaffold N50 of 17.22 Mb ( Table 2).

**Table 2.** Genome assembly statistics.

**Assembly name**	ilColMult2.hap1.1	ilColMult2.hap2.1
**Assembly accession**	GCA_965278185.1	GCA_965278165.1
**Assembly level**	chromosome	scaffold
**Span (Mb)**	482.72	504.67
**Number of chromosomes**	31	scaffold-level
**Number of contigs**	364	7 708
**Contig N50**	3.57 Mb	0.21 Mb
**Number of scaffolds**	124	5 268
**Scaffold N50**	17.22 Mb	14.26 Mb
**Longest scaffold length (Mb)**	22.73	-
**Sex chromosomes**	Z	-
**Organelles**	Mitochondrion: 17.63 kb	-

**Figure 3.**
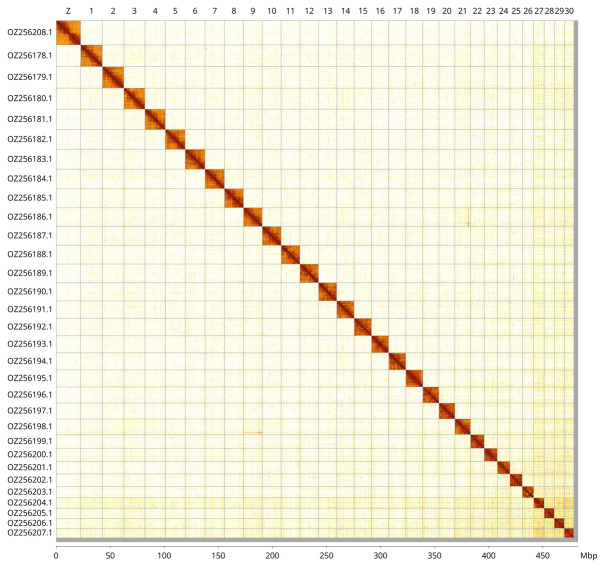
Hi-C contact map of the *Colostygia multistrigaria* genome assembly. Assembled chromosomes are shown in order of size and labelled along the axes, with a megabase scale shown below. The plot was generated using PretextSnapshot.

Most of the haplotype 1 assembly sequence (99.19%) was assigned to 31 chromosomal-level scaffolds, representing 30 autosomes and the Z sex chromosome. These chromosome-level scaffolds, confirmed by Hi-C data, are named according to size ( Figure 3; Table 3). The Z chromosome was identified based on BUSCO gene painting with ancestral Merian elements ( Wright *et al.*, 2024).

**Table 3.** Chromosomal pseudomolecules in the haplotype 1 genome assembly of *Colostygia multistrigaria* ilColMult2.

INSDC accession	Molecule	Length (Mb)	GC%
OZ256178.1	1	20.04	37.50
OZ256179.1	2	19.89	37.50
OZ256180.1	3	19.55	37.50
OZ256181.1	4	18.78	37.50
OZ256182.1	5	18.44	37
OZ256183.1	6	18.32	37
OZ256184.1	7	17.96	37
OZ256185.1	8	17.55	37
OZ256186.1	9	17.44	37.50
OZ256187.1	10	17.43	37.50
OZ256188.1	11	17.30	37
OZ256189.1	12	17.22	37
OZ256190.1	13	16.94	37.50
OZ256191.1	14	16.08	37.50
OZ256192.1	15	16.05	37
OZ256193.1	16	15.90	37
OZ256194.1	17	15.77	37.50
OZ256195.1	18	15.75	37.50
OZ256196.1	19	14.92	37.50
OZ256197.1	20	14.74	37.50
OZ256198.1	21	14.51	38
OZ256199.1	22	12.59	37.50
OZ256200.1	23	12.09	38
OZ256201.1	24	11.67	37.50
OZ256202.1	25	11.50	38
OZ256203.1	26	10.56	37.50
OZ256204.1	27	9.66	39
OZ256205.1	28	9.32	38
OZ256206.1	29	9.23	38.50
OZ256207.1	30	8.87	38.50
OZ256208.1	Z	22.73	37

The mitochondrial genome was also assembled (length 17.63 kb, OZ256209.1). This sequence is included as a contig in the multifasta file of the genome submission and as a standalone record.

### Assembly quality metrics

**Figure 4.**
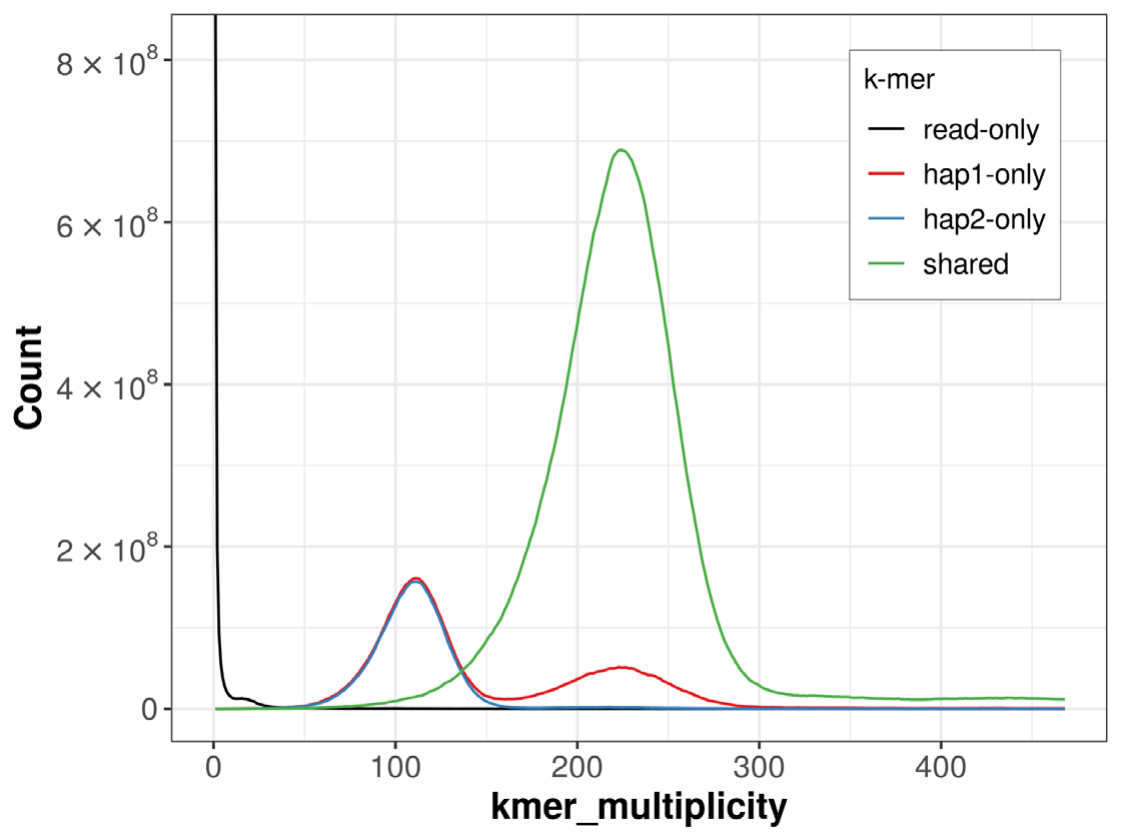
Evaluation of *k*-mer completeness using MerquryFK. This plot illustrates the recovery of *k*-mers from the original read data in the final assemblies. The horizontal axis represents *k*-mer multiplicity, and the vertical axis shows the number of *k*-mers. The black curve represents *k*-mers that appear in the reads but are not assembled. The green curve corresponds to *k*-mers shared by both haplotypes, and the red and blue curves show *k*-mers found only in one of the haplotypes.

For haplotype 1, the estimated QV is 61.3, and for haplotype 2,59.2. When the two haplotypes are combined, the assembly achieves an estimated QV of 60.1. The *k*-mer completeness is 83.69% for haplotype 1, 78.87% for haplotype 2, and 99.76% for the combined haplotypes ( Figure 4).

**Figure 5.**
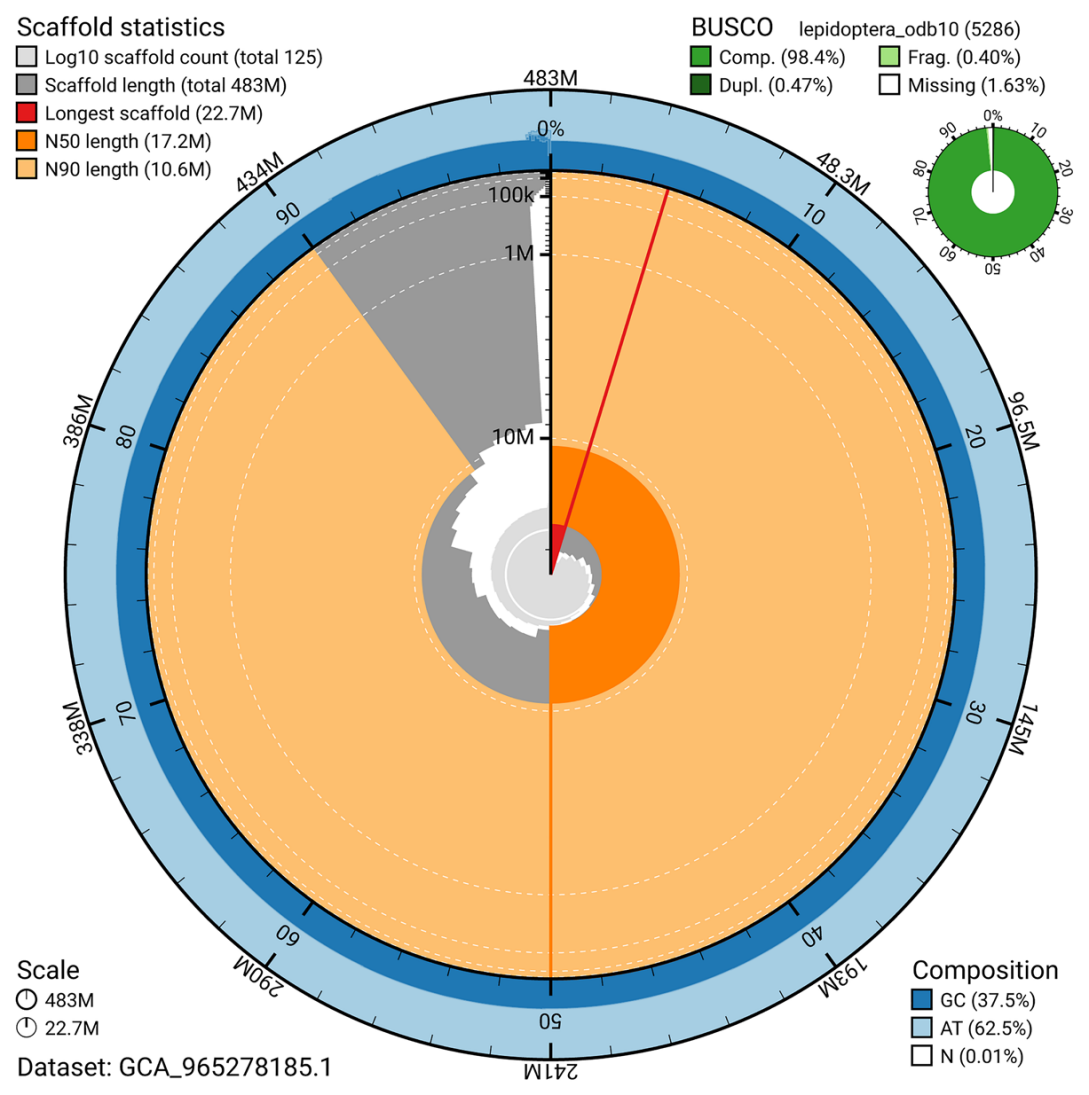
Assembly metrics for ilColMult2.hap1.1. The BlobToolKit snail plot provides an overview of assembly metrics and BUSCO gene completeness. The circumference represents the length of the whole genome sequence, and the main plot is divided into 1 000 bins around the circumference. The outermost blue tracks display the distribution of GC, AT, and N percentages across the bins. Scaffolds are arranged clockwise from longest to shortest and are depicted in dark grey. The longest scaffold is indicated by the red arc, and the deeper orange and pale orange arcs represent the N50 and N90 lengths. A light grey spiral at the centre shows the cumulative scaffold count on a logarithmic scale. A summary of complete, fragmented, duplicated, and missing BUSCO genes in the set is presented at the top right. An interactive version of this figure can be accessed on the BlobToolKit viewer.

**Figure 6.**
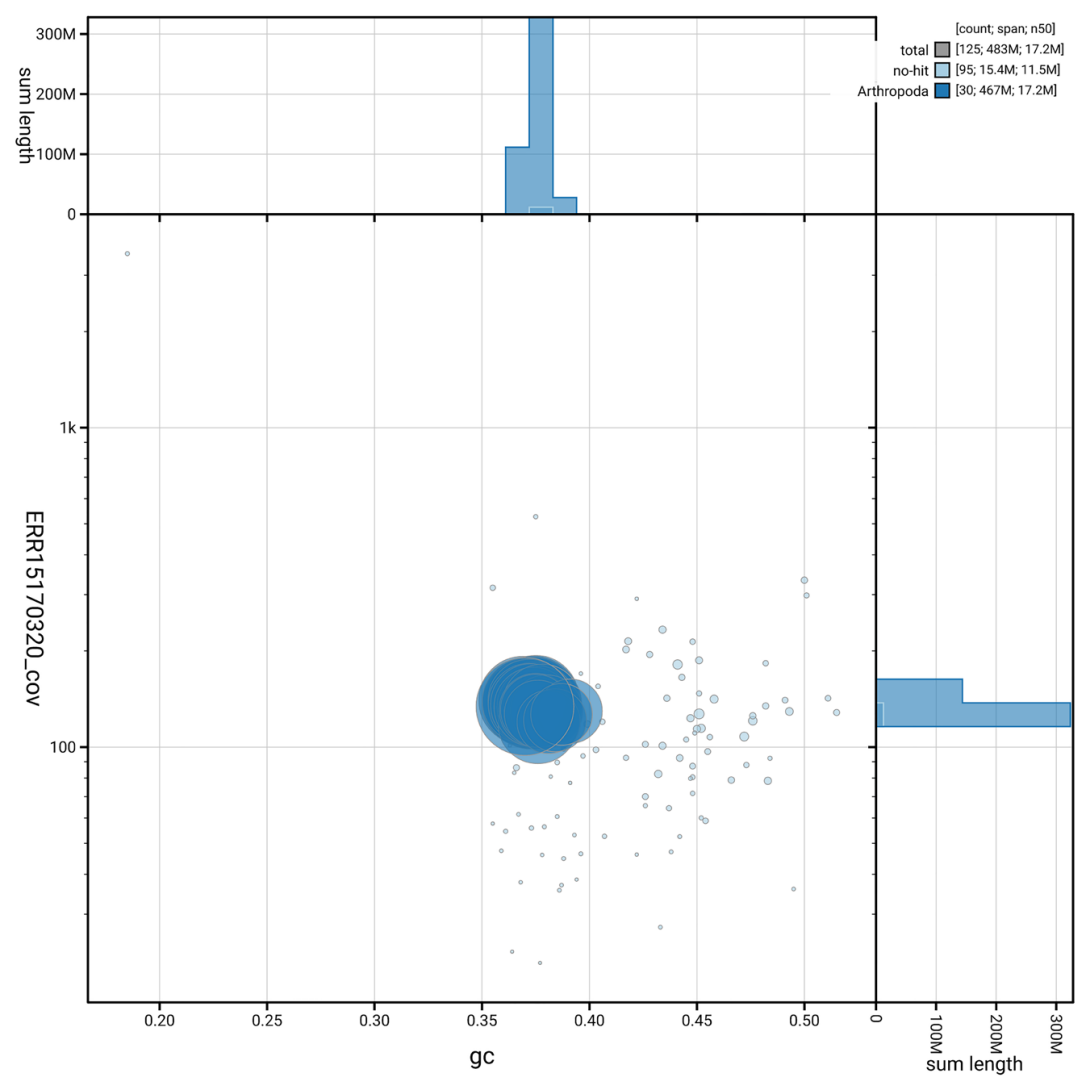
BlobToolKit GC-coverage plot for ilColMult2.hap1.1. Blob plot showing sequence coverage (vertical axis) and GC content (horizontal axis). The circles represent scaffolds, with the size proportional to scaffold length and the colour representing phylum membership. The histograms along the axes display the total length of sequences distributed across different levels of coverage and GC content. An interactive version of this figure is available on the BlobToolKit viewer.

BUSCO analysis using the lepidoptera_odb10 reference set ( *n* = 5 286) identified 98.4% of the expected gene set (single = 97.9%, duplicated = 0.5%) in haplotype 1. For haplotype 2, BUSCO v.5.7.1 analysis identified 91.3% of the expected gene set (single = 87.5%, duplicated = 3.9%). The snail plot in Figure 5 summarises the scaffold length distribution and other assembly statistics for haplotype 1. The blob plot in Figure 6 shows the distribution of scaffolds by GC proportion and coverage for haplotype 1.

Table 4 lists the assembly metric benchmarks adapted from Rhie *et al.* (2021) and the Earth BioGenome Project Report on Assembly Standards September 2024. The EBP metric, calculated for the haplotype 1, is **6.C.Q61**, meeting the recommended reference standard.

**Table 4.** Earth Biogenome Project summary metrics for the *Colostygia multistrigaria* assembly.

Measure	Value	Benchmark
EBP summary (haplotype 1)	6.C.Q61	6.C.Q40
Contig N50 length	3.57 Mb	≥ 1 Mb
Scaffold N50 length	17.22 Mb	= chromosome N50
Consensus quality (QV)	Haplotype 1: 61.3; haplotype 2: 59.2; combined: 60.1	≥ 40
*k*-mer completeness	Haplotype 1: 83.69%; Haplotype 2: 78.87%; combined: 99.76%	≥ 95%
BUSCO	C:98.4% [S:97.9%; D:0.5%]; F:0.4%; M:1.2%; n:5 286	S > 90%; D < 5%
Percentage of assembly assigned to chromosomes	99.19%	≥ 90%

### Wellcome Sanger Institute – Legal and Governance

The materials that have contributed to this genome note have been supplied by a Darwin Tree of Life Partner. The submission of materials by a Darwin Tree of Life Partner is subject to the **‘Darwin Tree of Life Project Sampling Code of Practice’**, which can be found in full on the Darwin Tree of Life website. By agreeing with and signing up to the Sampling Code of Practice, the Darwin Tree of Life Partner agrees they will meet the legal and ethical requirements and standards set out within this document in respect of all samples acquired for, and supplied to, the Darwin Tree of Life Project. Further, the Wellcome Sanger Institute employs a process whereby due diligence is carried out proportionate to the nature of the materials themselves, and the circumstances under which they have been/are to be collected and provided for use. The purpose of this is to address and mitigate any potential legal and/or ethical implications of receipt and use of the materials as part of the research project, and to ensure that in doing so we align with best practice wherever possible. The overarching areas of consideration are:

•    Ethical review of provenance and sourcing of the material

•    Legality of collection, transfer and use (national and international)

Each transfer of samples is further undertaken according to a Research Collaboration Agreement or Material Transfer Agreement entered into by the Darwin Tree of Life Partner, Genome Research Limited (operating as the Wellcome Sanger Institute), and in some circumstances, other Darwin Tree of Life collaborators.

## Data Availability

European Nucleotide Archive: Colostygia multistrigaria (mottled grey). Accession number PRJEB84117. The genome sequence is released openly for reuse. The *Colostygia multistrigaria* genome sequencing initiative is part of the Darwin Tree of Life Project (PRJEB40665), Sanger Institute Tree of Life Programme (PRJEB43745) and Project Psyche (PRJEB71705). All raw sequence data and the assembly have been deposited in INSDC databases. The genome will be annotated using available RNA-Seq data and presented through the Ensembl pipeline at the European Bioinformatics Institute. Raw data and assembly accession identifiers are reported in Table 1 and Table 2. Production code used in genome assembly at the WSI Tree of Life is available at https://github.com/sanger-tol. Table 5 lists software versions used in this study.

## Author information

Contributors are listed at the following links:

- Members of the University of Oxford and Wytham Woods Genome Acquisition Lab
- Members of the Darwin Tree of Life Barcoding collective
- Members of the Wellcome Sanger Institute Tree of Life Management, Samples and Laboratory team
- Members of Wellcome Sanger Institute Scientific Operations – Sequencing Operations
- Members of the Wellcome Sanger Institute Tree of Life Core Informatics team
- Members of the Tree of Life Core Informatics collective
- Members of the Darwin Tree of Life Consortium
