# Supplementary material for: The genome sequence of the Mottled Grey, Colostygia multistrigaria (Haworth, 1809) (Lepidoptera: Geometridae)
Source: Wellcome Open Res. 2025 Dec 23;10:697. [Version 1] doi: 10.12688/wellcomeopenres.25410.1 (PMC12800613; doi:10.12688/wellcomeopenres.25410.1)
Supplement: Peer review history

# Reviewer report: Reviewer response for version 1

**DOI:** 10.21956/wellcomeopenres.27996.r144524

**Published:** 2026-01-29

**Authors:** Shangzhe Zhang

**Affiliations:** 1 University of St Andrews Library, St Andrews, Scotland, UK

The data note of genome sequence and assembly of the Mottled Grey is well presented and would benefit the society as a part of ToL project. I have only a few concerns: 

 1. The sample ilColMult1 lacks clarification of sex. 

 2. I would suggest a further discussion on the big difference between haplotype assemblies, for example, contig N50 (3.57Mb vs. 0.21Mb). Does this represent a failed phasing using Hi-C data?

Are sufficient details of methods and materials provided to allow replication by others?

Yes

Is the rationale for creating the dataset(s) clearly described?

Yes

Are the datasets clearly presented in a useable and accessible format?

Yes

Are the protocols appropriate and is the work technically sound?

Yes

Reviewer Expertise:

Genomics, Evolutionary biology

I confirm that I have read this submission and believe that I have an appropriate level of expertise to confirm that it is of an acceptable scientific standard.

# Reviewer report: Reviewer response for version 1

**DOI:** 10.21956/wellcomeopenres.27996.r144522

**Published:** 2026-01-13

**Authors:** Michael Hiller

**Affiliations:** 1 Senckenberg Research Institute, Frankfurt, Germany

This data note describes another high-quality reference genome of a European lepidoptera. 

 I have three comments: 

 1) If a second individual was used for HiC data generation, I wonder how a haplotype-resolved assembly can be generated, as this typically requires both HiFi and HiC data coming from the same individual. I wonder how sequence variation and potential genome structure difference between both individuals were handled. There is also a large difference in contig N50 (3.57 vs 0.21 Mb) between the haplotypes. 

 2) Please mention which kit was used for the ULI lib prep; likely the standard PacBio kit before they released the Ampli-Fy kit? 

 3) typo in "The The specimens were collected "

Are sufficient details of methods and materials provided to allow replication by others?

Yes

Is the rationale for creating the dataset(s) clearly described?

Yes

Are the datasets clearly presented in a useable and accessible format?

Yes

Are the protocols appropriate and is the work technically sound?

Yes

Reviewer Expertise:

comparative genomics, genome assembly

I confirm that I have read this submission and believe that I have an appropriate level of expertise to confirm that it is of an acceptable scientific standard.

# Reviewer report: Reviewer response for version 1

**DOI:** 10.21956/wellcomeopenres.27996.r143274

**Published:** 2026-01-09

**Authors:** Shinya Komata

**Affiliations:** 1 Institute of Science Tokyo, Meguro-ku, Tokyo, Japan

This study presents a genome assembly of *Colostygia multistrigaria*, a geometrid moth, including Hi-C–based scaffolding. Both data generation and analyses are of sufficient quality. The methods are described in adequate detail, and there are no major concerns in this regard.

 If any clarification were to be added, it might be helpful to comment on the relatively lower contiguity of haplotype 2, rather than its base-level accuracy. In particular, haplotype 2 shows a much higher number of contigs and an extremely small contig N50 compared with haplotype 1. In addition, there is a noticeable discrepancy between the estimated genome size (446.5 Mb) and the assembly spans (hap1: 482.72 Mb; hap2: 504.67 Mb), which is likely expected for a phased diploid assembly but could benefit from a brief explanation to reassure readers that this does not reflect an assembly problem.

Are sufficient details of methods and materials provided to allow replication by others?

Yes

Is the rationale for creating the dataset(s) clearly described?

Yes

Are the datasets clearly presented in a useable and accessible format?

Yes

Are the protocols appropriate and is the work technically sound?

Yes

Reviewer Expertise:

Evolutionary biology; genomics; butterfly

I confirm that I have read this submission and believe that I have an appropriate level of expertise to confirm that it is of an acceptable scientific standard.
